# Prevalence and patterns of anti-angiogenic treatment discontinuation in neovascular age-related macular degeneration—insights from health claims data

**DOI:** 10.1038/s41433-026-04333-3

**Published:** 2026-02-24

**Authors:** Nicolas S. Bodmer, Livia Faes, Nico Gross, Lucas M. Bachmann, Carola Huber, Markus Näpflin, Martin K. Schmid

**Affiliations:** 1 University of Zurich Epidemiology, Biostatistics & Prevention Institute (EBPI), Zurich, Switzerland; 2 Medignition Inc. Research Consultants Zurich, Zurich, Switzerland; 3 Institute of Health Policy, Management and Evaluation, Dalla Lana School of Public Health, University of Toronto, Toronto, ON Canada; 4 Cantonal Hospital Winterthur, Winterthur, Switzerland; 5 Moorfields Eye Hospital NHS Foundation Trust, London, UK; 6 Eye Clinic, Cantonal Hospital Lucerne, Lucerne, Switzerland; 7 Department of Health Sciences, Helsana Group, Zürich, Switzerland; 8 Department of Health Sciences and Medicine, University of Lucerne, Lucerne, Switzerland

**Keywords:** Health services, Medical research

## Abstract

**Purpose:**

To assess patterns and institutional variability of anti-angiogenic treatment discontinuation in neovascular age-related macular degeneration (nAMD) within Switzerland, and to examine the association of digital home monitoring with treatment persistence.

**Methods:**

Retrospective observational analysis combined nationwide health insurance claims data (Helsana, *n* = 4840) and clinical data from a major tertiary centre (LUKS, *n* = 1932; nAMD subset *n* = 1387). Discontinuation was defined as no further injections for 31–365 days after the last intravitreal injection (IVI) and classified as supervised ( ≥ 1 follow-up visit in this period) or unsupervised (no follow-up visits in this period). Variability was evaluated among the 20 largest providers and across seven regions. Digital home monitoring’s impact on non-persistence was assessed in the clinical cohort.

**Results:**

Substantial heterogeneity was observed. Supervised discontinuation ranged from 20.7 to 45.7% (pooled proportion 32.0%, 95% confidence interval [CI] 30.2–33.9%) and unsupervised from 10.9 to 25.7% (pooled proportion 18.2%, 95%CI 16.8–19.8%) across centres. In the LUKS cohort, 1-year persistence was 93.7%, dropping to 72.7% by year 4, with unsupervised discontinuation rising from 3.0 to 12.5%. In 100 patients using voluntary digital home monitoring non-persistence was less likely (adjusted OR 0.59, 95%CI 0.35–0.98); among those who discontinued, home-monitoring users were more likely to remain under supervision (74% vs 54%).

**Conclusions:**

Treatment discontinuation in nAMD is frequent and demonstrates striking institutional and regional variability. The heterogeneity in discontinuation patterns suggests that treatment persistence may be modifiable through targeted, systematic, and patient-centred interventions. Digital home monitoring may be a promising strategy to support engagement and reduce non-persistence.

## Introduction

Exudative neovascular age-related macular degeneration (nAMD) represents a chronic, progressive chorioretinal condition and the leading cause of irreversible central vision loss in high-income nations [1]. The pathophysiology of nAMD involves complex molecular cascades, including upregulation of vascular endothelial growth factor (VEGF), angiopoietin-2 (Ang2), and other pro-angiogenic mediators that drive choroidal neovascularisation, vascular permeability, and subsequent photoreceptor damage [2, 3]. The introduction of intravitreal anti-angiogenic therapy has fundamentally transformed disease outcomes, with pivotal randomised controlled trials demonstrating visual stabilisation and improvement in the majority of treated patients [4–6].

Despite established therapeutic efficacy, real-world visual outcomes consistently fall short of clinical trial results [7]. Treatment discontinuation represents a critical barrier to sustained visual benefit, encompassing two distinct phenomena: non-persistence (patient-driven cessation due to barriers such as access limitations, comorbidities, psychosocial factors or treatment burden) and planned discontinuation (physician-initiated cessation based on perceived treatment futility or apparent disease quiescence), as defined by Okada et al.‘s consensus framework [8]. A systematic review of 409,215 patients found that patient-led non-adherence ranged from 17.5 to 35.0% depending on definition, with overall pooled non-persistence of 30.0% [9]. While physician-initiated treatment exit strategies remain highly controversial in AMD management due to high recurrence rates upon cessation, patient-driven non-persistence may represent a more significant modifiable barrier to sustained visual benefit [10–12].

Post hoc analyses from trials and real-world evidence demonstrate that treatment adherence is associated with superior visual outcomes. In a secondary analysis of the Comparison of Age-Related Macular Degeneration Treatment Trial (CATT), visit adherence to scheduled anti-VEGF injections was strongly associated with better visual acuity maintenance, with substantial vision loss observed in patients who miss appointments or discontinue treatment [13]. In real-world cohorts and registries, non-persistent patients experienced significantly greater visual acuity decline compared to those who persisted with treatment, with non-persistent patients also demonstrating a substantially increased risk of developing subfoveal fibrosis [14, 15].

Emerging evidence suggests that digital home monitoring technologies may improve treatment persistence in chronic retinal diseases. Hyperacuity-based applications enable patients to detect vision changes between visits, potentially maintaining engagement with their treatment regimen [16]. Given that enhanced patient monitoring may represent a scalable intervention to improve treatment continuity, we examine the association between home monitoring utilisation and treatment persistence in our cohort.

Importantly, Switzerland’s universal healthcare coverage provides a unique epidemiological context for examining treatment discontinuation patterns without the confounding influence of insurance-related access barriers that characterise other healthcare systems. The present investigation therefore utilises comprehensive Swiss health insurance claims data to quantify treatment discontinuation prevalence and patterns in patients receiving anti-angiogenic therapy for nAMD and related conditions.

We hypothesise that treatment discontinuation remains prevalent within Switzerland’s universal healthcare system, with significant institutional variation reflecting both inconsistent physician adherence to continuous treatment principles and patient-driven non-persistence due to systemic barriers, and that enhanced patient engagement through digital monitoring technologies may represent a modifiable factor associated with improved treatment persistence.

## Methods

### Study design

This was a retrospective, observational study combining two complementary datasets: nationwide health insurance claims data from Helsana AG (Switzerland’s largest private health insurer) and detailed clinical data from the Cantonal Hospital Lucerne (LUKS), the largest tertiary referral centre for medical retina in Switzerland. This dual approach enabled comprehensive analysis of treatment discontinuation patterns in prevalent anti-VEGF treatment cohorts at both population and institutional levels.

### Data sources and study populations

#### LUKS clinical dataset

**Fig. 1:**
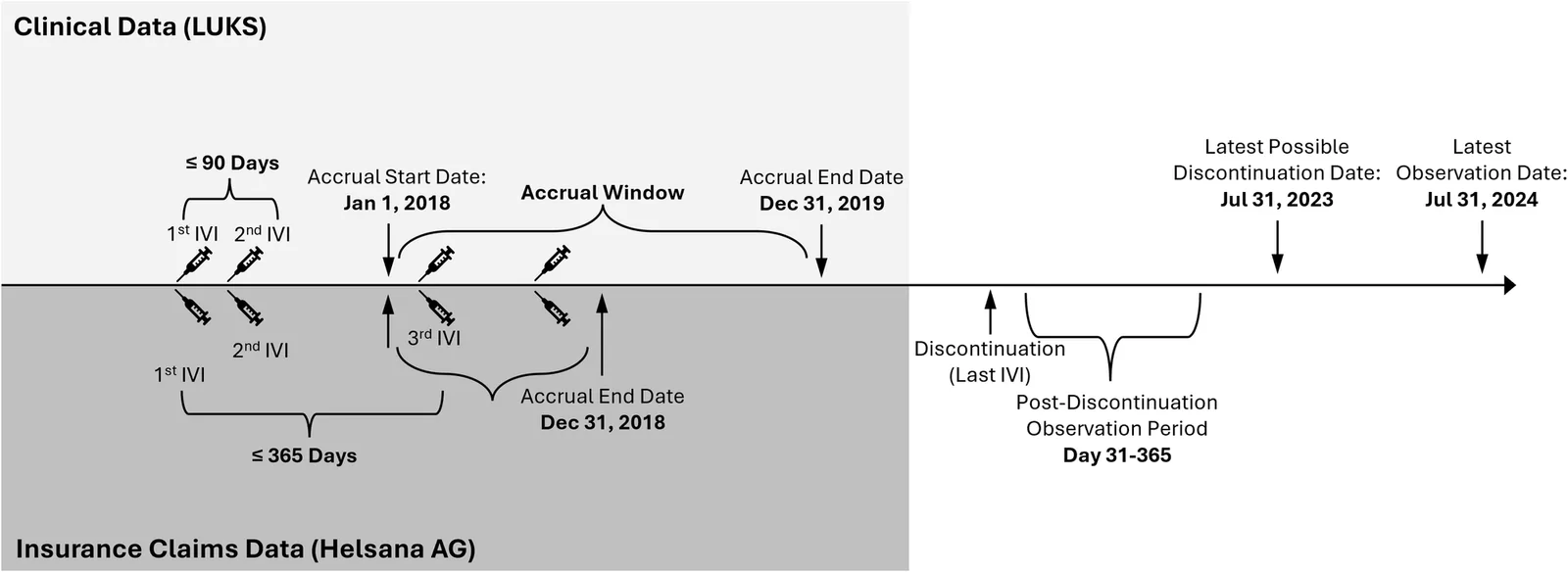
Accrual periods and inclusion criteria for the clinical (Cantonal Hospital Lucerne) and Insurance Claims (Helsana AG) cohorts. This figure illustrates the respective time windows, patient selection processes, and key criteria applied to construct each study population.

The LUKS dataset (prevalent cohort) provided detailed clinical information from a single tertiary centre. Inclusion criteria required at least one intravitreal injection (IVI) between January 1, 2018, and December 31, 2019, with ≥4 injections overall and at least two injections within 90 days of the first injection to ensure active treatment initiation. The ≥4 injection criterion ensured analysis of patients who completed at least a standard loading phase plus one additional treatment, while the 90-day window captured the typical loading phase interval for anti-angiogenic therapy, distinguishing patients who initiated sustained treatment from those who received only exploratory or discontinued early therapy. For patients with bilateral disease, the second eye was selected if it received ≥4 injections; otherwise, the first eye was included. The index date was defined as the date of the first IVI documented in the clinical information system, which generally marked the initiation of treatment. Diagnosis of nAMD was established by manual review of the clinical medical record diagnosis fields. Please refer to Fig. 1 for an overview of patient selection and accrual window, and to Supplementary Material 1 for details on the extracted clinical variables.

Within the LUKS cohort, a subset of patients participated in voluntary digital hyperacuity home monitoring using a CE-marked mobile application (*Alleye, Oculocare Medical AG, Switzerland*). Participation was voluntary and provided at no cost to patients. The application enabled patients to monitor their central vision using hyperacuity-based tests performed on smartphones or tablets, with the goal of early detection of vision changes between clinic visits. (https://www.alleye.io/)

Home monitoring status was retrospectively abstracted from clinical records and classified as a binary variable: patients were designated as either *users* (engaged with home monitoring at any time during the observation period) or *non-users* (never engaged). Among users, those who had monitoring activity within 30 days before treatment discontinuation were further classified as *active users*.

#### Helsana claims dataset

The insurance claims dataset encompassed a nationwide sample of patients covered by Helsana AG, Switzerland’s largest private health insurer, accounting for roughly 15% of the national population with strong representation in German-speaking regions. The database provided geographically broad coverage across Switzerland and captured care from both large ophthalmology centres and smaller practices, thereby reflecting the country’s multilingual and diverse healthcare setting. The final dataset captured comprehensive details of both out- and inpatient care, including IVI, and clinical visits to private practices as well as tertiary centres.

Inclusion criteria required patients to have had continuous insurance coverage by Helsana AG during the year 2018 and have received at least one IVI between January 1 and December 31, 2018, with ≥4 injections overall and at least three injections within 365 days from baseline. The 4-injection threshold was selected to distinguish patients who progressed beyond initial treatment trials and received sustained therapy, as sporadic or minimal treatment exposure ( ≤ 3 injections) likely reflects either treatment discontinuation during the loading phase or experimental treatment that was quickly abandoned. The index date was defined as the first IVI course in which patients received ≥3 injections within 365 days recorded in the Helsana claims database (vs a 90-day threshold used in the clinical dataset). This strategy was chosen to ensure that treatment initiation could be reliably identified within Helsana claims data.

To ensure comparability and follow-up across cases, the analysis included only patients insured within a single calendar year, using a 1-year accrual period distinct from the approach in the clinical dataset. Due to limitations of claims data, which do not capture eye-specific information, all analyses were performed at the patient level. Patients were classified as having AMD if they did not have diabetes (identified according to standard claims-based criteria) though differentiation from other retinal vascular diseases was not possible. See Fig. 1 for patient selection and accrual details, and Supplementary Material 2 for full cohort definitions and insurance variable descriptions.

#### Linkage (Subset Helsana-insured patients at LUKS)

To enable validation of diagnoses and assess concordance between clinical and insurance data, clinical records for Helsana-insured patients treated at LUKS were linked with their corresponding health insurance claims. Privacy was protected by employing the Privacy Preserving Probabilistic Record Linkage (P3RL) method: identifying information (including name, date of birth, gender, and zip code) was encrypted and remained securely within each institution [17]. The actual linkage was performed by an independent trusted centre using G-LINK probabilistic record linkage software developed by Statistics Canada [18]. Only pseudonymous site identifiers were included in the resulting link table, which was then utilised for subsequent analyses.

### Key definitions

#### Treatment discontinuation

Treatment discontinuation was defined as the date of the last recorded IVI with a 30-day grace period accounting for normal scheduling variation, documentation and billing errors. In the subsequent observation window of 31–365 days without further injections, a critical distinction was made between “supervised” and “unsupervised” treatment discontinuation:

Supervised discontinuation: patients who, following their final injection, maintained at least one ophthalmological monitoring visit during the observation window suggesting continued clinical engagement despite treatment cessation.

Unsupervised discontinuation: patients who received no further ophthalmological care during the observation window, representing complete disengagement and placing these individuals at highest risk for undetected disease reactivation and irreversible vision loss. In the insurance claims dataset, we were able to distinguish unsupervised discontinuation due to death from unsupervised discontinuation for other reasons based on recorded vital status, whereas in the LUKS clinical cohort, we could not reliably make this distinction because vital status was not available.

While supervised discontinuation may reflect a mix of physician-led planned treatment cessation and patient-driven decisions not to continue active therapy, unsupervised discontinuation is unlikely to result from physician recommendation and may thus serve as a closer proxy for true patient-driven non-persistence. Notably, our definition employs a 12-month window as opposed to the 6-month window proposed by Okada and co-workers [8].

### Outcome measures

The primary outcome was the proportion of patients experiencing supervised or unsupervised treatment discontinuation during the observation period. This was evaluated in both the clinical dataset (LUKS) and the national claims dataset (Helsana), with stratification by the 20 largest treatment centres and by smaller clinics aggregated by region (*see* Supplementary Material 3*for details*). Secondary outcomes included yearly proportions (up to 4 years from index date) of supervised and unsupervised discontinuation in the LUKS cohort, as well as the extent of home monitoring engagement within a LUKS patient subgroup.

### Statistical analysis

Patient demographics and baseline clinical characteristics were summarised as mean (standard deviation, SD) for normally distributed variables and as median (interquartile range, IQR) for non-normally distributed variables. Centre- and region-specific proportions of supervised and unsupervised treatment discontinuation were depicted in forest plots with 95% confidence intervals (CI), calculated using the Wilson score method; for centres with very low patient numbers or extreme outcomes, exact (Clopper–Pearson) intervals were provided. In the home monitoring analysis, the association between treatment discontinuation and home monitoring engagement was evaluated using a generalised linear model with a binomial distribution, adjusting for age, sex, baseline visual acuity, and baseline central retinal thickness. Associations were reported as odds ratios with corresponding 95% CIs. All statistical analyses were conducted using R version 4.3.3 (R Foundation for Statistical Computing, Vienna, Austria).

### Ethical considerations

In the clinical dataset, only patients who had provided general consent for the secondary use of their health data for research purposes were included. For the insurance claims dataset, anonymised data were analysed in accordance with federal regulations, which do not require individual informed consent. For the subset of LUKS patients whose records were linked with health insurance claims, an exemption under Article 34 of the Swiss Human Research Act (HRA) was formally obtained. The study adhered to the principles of the Declaration of Helsinki and applicable Swiss data protection laws. Ethical approval was granted in accordance with national regulatory requirements (BASEC No. 2022-01047).

## Results

### Patient characteristics

This investigation included 4840 patients from the nationwide Helsana insurance claims database and 1932 patients from the LUKS clinical cohort, constituting one of the largest real-world analyses of anti-angiogenic treatment discontinuation. The mean age was 77 years (SD 10) in the insurance claims cohort and 74 years (SD 11) in the clinical cohort. Female representation was comparable between cohorts, with proportions of 61% in the claims and 57% in the clinical group.

In the LUKS clinical cohort, 1387 patients (72%) were treated for nAMD, 239 (12%) for diabetic macular oedema (DMO), and 306 (16%) for other retinal pathologies. The claims database classified a higher proportion of cases as DMO (23%), reflecting the use of an algorithm that attributed all intravitreal injections in diabetic patients to DMO. As a result, differentiation between nAMD and other retinal vascular disorders was not feasible in the claims data. Nonetheless, clinical cohort data suggested that, after exclusion of DMO, over 82% of anti-angiogenic therapy recipients were treated for nAMD, with other indications representing only a minority.

Among the subset of Helsana-insured patients treated at LUKS, concordance between clinical and claims datasets was high. Detailed patient characteristics are available in Table 1; Supplementary Table 1 provides additional clinical characteristics of the LUKS cohort, and Supplementary Table 2 presents a centre- and region-level overview of patient features from the claims dataset.

**Table 1:** Patient characteristics.

Characteristic	LUKS *n* = 1932	Health insurance claims *n* = 4840
**Age, mean (SD)**	74 (11)	77 (10)
**Gender**		
Female, n (%)	1102 (57%)	2951 (61%)
Male, n (%)	830 (43%)	1889 (39%)
**Bilateral affection, n (%)**^**a**^	863 (45%)	n.a.
**Diagnosis**		
AMD, n (%)	1387 (72%)	3722 (77%)^b^
Other retinal diseases, n (%)	306 (16%)	
DMO, n (%)	239 (12%)	1118 (23%)
**Treatment duration in days, mean (SD)**^c^	2135 (1370)	1811 (896)

*AMD* age-related macular degeneration, *DMO* diabetic macular oedema, *SD* standard deviation, *LUKS* Cantonal Hospital Lucerne.

^a^Received treatment for both eyes. Based on insurance claims not possible to identify.

^b^Based on insurance claims, the distinction between AMD vs. other retinal diseases was not possible. Patients with diabetes were assumed to receive intravitreal injection for DMO.

^c^Treatment duration was calculated from first injection to last injection.

### Treatment discontinuation patterns and profound institutional variability

**Fig. 2:**
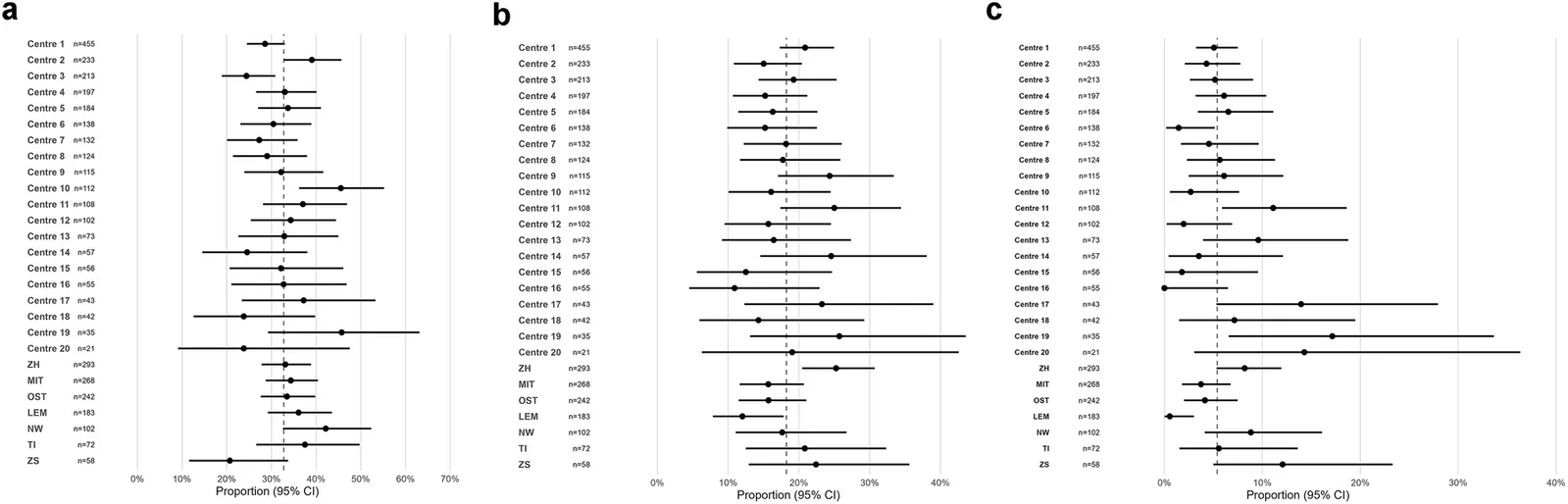
Regional Variability in Treatment Discontinuation. **a** Institutional and Regional Variability in Treatment Discontinuation in Proportions: Supervised Discontinuation in Neovascular Age-related Macular Degeneration Patients. Forest plot displaying supervised discontinuation proportions for therapy of neovascular age-related macular degeneration (AMD) across major Swiss ophthalmology providers and aggregated regions. Each site is represented by the proportion of supervised discontinuation with 95% confidence intervals (CI); “n” indicates the number of AMD patients per centre or region. Regional abbreviations: LEM = Région lémanique (Lake Geneva region); MIT = Espace Mittelland (Central Mittelland); NW = Nordwestschweiz (Northwestern Switzerland); OST = Ostschweiz (Eastern Switzerland); TI = Ticino; ZH = Zürich; ZS = Zentralschweiz (Central Switzerland). **b** Institutional and Regional Variability in Treatment Discontinuation Proportions: Unsupervised Discontinuation in Neovascular Age-related Macular Degeneration Patients. Forest plots illustrate the proportions of unsupervised discontinuation among patients with neovascular age-related macular degeneration (nAMD) across Swiss ophthalmology centres and aggregated regions. Each point represents a site or region, with proportions displayed alongside 95% confidence interval (CI) error bars. “n” indicates the number of patients per centre or region. Regional abbreviations: LEM = Région lémanique (Lake Geneva region); MIT = Espace Mittelland (Central Mittelland); NW = Nordwestschweiz (Northwestern Switzerland); OST = Ostschweiz (Eastern Switzerland); TI = Ticino; ZH = Zürich; ZS = Zentralschweiz (Central Switzerland). **c** Institutional and Regional Variability in Unsupervised Discontinuation Proportions among Neovascular Age-related Macular Degeneration Patients Surviving Post-Discontinuation Follow-up. Forest plots display the proportions of unsupervised discontinuation among patients who survived the post-discontinuation observation period, with each centre or region shown as a point estimate flanked by 95% confidence interval (CI) error bars. The value “n” specifies the number of patients for each site or region. Regions: LEM = Région lémanique (Lake Geneva region); MIT = Espace Mittelland (Central Mittelland); NW = Nordwestschweiz (Northwestern Switzerland); OST = Ostschweiz (Eastern Switzerland); TI = Ticino; ZH = Zürich; ZS = Zentralschweiz (Central Switzerland).

Analysis of the 20 largest service providers and 7 aggregated geographic regions within the national Helsana dataset demonstrated marked institutional variation in treatment discontinuation proportions. Supervised discontinuation proportions ranged widely across centres, from 20.7 to 45.7% (pooled proportion 32.0%, 95% CI 30.2–33.9%), while unsupervised discontinuation proportions varied between 10.9% and 25.7% (pooled proportion 18.2%, 95% CI 16.8–19.8%). Restricting the analysis to unsupervised discontinuation among patients surviving the 365-day observation period still yielded proportions as high as 17.1% (pooled proportion 5.4%, 95% CI 4.6-6.4%). Notably, this heterogeneity was unrelated to provider case volume, implicating substantial differences in care continuity and practice patterns across institutions. Changes in health insurance coverage were unlikely to confound these results, as only 1.5% of patients switched insurers during the study period. Corresponding forest plots for supervised, unsupervised, and unsupervised discontinuation excluding death are presented in Fig. 2a–c.

### Long-term treatment persistence in AMD patients

Longitudinal assessment of the LUKS AMD cohort (*n* = 1387) revealed a marked decline in treatment persistence over time. Persistence proportions decreased from 93.7% at one year to 72.7% by year four, highlighting a substantial trend toward long-term treatment discontinuation. Notably, the incidence of unsupervised discontinuation rose from 3.0% in the first year to 12.5% by year four, identifying a growing subgroup of patients at elevated risk for silent disease reactivation and irreversible vision loss. Supervised discontinuation also increased (from 3.4% to 14.8%), indicating that, while some patients maintained follow-up despite stopping therapy, a considerable fraction ultimately disengaged entirely from ophthalmologic care.

### Digital home monitoring and enhanced treatment persistence

Among the 1387 patients included in the analysis, 100 individuals (7.2%) consistently engaged in hyperacuity home monitoring throughout the observation period. Compared to *non-users*, individuals utilising home monitoring were significantly younger (mean age 73 years [SD 8] vs. 77 years [SD 9]; *p* < 0.001), received a higher mean number of IVI (52 [SD 32] vs. 38 [SD 22]; *p* < 0.001), and exhibited superior best-corrected visual acuity at baseline (71 [SD 13] vs. 63 [SD 17] ETDRS letters; *p* < 0.001). Central retinal thickness was comparable between users and non-users (361 µm [SD 101] vs. 381 µm [SD 124]; *p* = 0.3). Comprehensive baseline characteristics for AMD patients at LUKS are presented in Supplementary Table 1. In adjusted analyses, recent engagement in hyperacuity home monitoring (within 30 days of the last IVI) was associated with significantly lower odds of treatment discontinuation (odds ratio 0.589, 95% CI: 0.346–0.979; *p* < 0.05). Moreover, among those who discontinued therapy, patients using home monitoring were substantially more likely to maintain supervised follow-up rather than disengage entirely from care (74% vs. 54%, *p* < 0.05).

## Discussion

### Main Findings

This nationwide Swiss analysis reveals that patient-driven treatment discontinuation in nAMD is a significant and underrecognised challenge in real-world practice, with profound institutional variability suggesting substantial quality gaps in care delivery. Our findings demonstrate that unsupervised discontinuation representing complete disengagement from ophthalmologic care, increased dramatically from 3.0% at year 1 to 12.5% by year 4. Most importantly, we identified striking heterogeneity across the 20 largest treatment centres, with unsupervised discontinuation proportions varying from 0.0% (95% CI 0–6.5%) to 17.1% (95% CI 6.6–33.6%) between institutions, indicating that treatment persistence may be substantially modifiable through systematic quality improvement initiatives. Our finding that unsupervised discontinuation proportions were highly variable across institutions likely reflects differences in patient-driven non-persistence, since such complete disengagement from care is rarely recommended by physicians. The more stringent 12-month window we applied, compared to the Okada definition, should be considered when comparing across studies [8].

### Results in context with the existing evidence

Contemporary management employs treat-and-extend regimens that safely prolong injection intervals to 12–16 weeks while maintaining disease control. However, systematic exit strategies remain highly controversial. Despite some expert protocols recommending suspension after consecutive 16-week stable intervals, real-world studies report recurrence proportions escalating to 79% at 5 years [19]. Critically, early reactivation is often asymptomatic, with only 40% of patients detecting recurrence via visual symptoms [20]. Each reactivation event correlates with 2–4 letter visual acuity loss and four-fold increased risk of subretinal fibrosis [13, 14, 19–21]. Given these findings, the high proportions of discontinuation identified in our study are especially concerning, as they may heighten the risk of unrecognised disease recurrence and irreversible vision loss in the real-world patient population [22, 23]. The substantial differences in formal discontinuation proportions observed across institutions may reflect considerable heterogeneity in how Swiss AMD care providers interpret and implement contemporary treatment guidelines. These findings suggest that, in the absence of consensus, variable guideline adoption could contribute to inconsistent patient management and potentially impact long-term visual outcomes.

Our Swiss findings align with the lower end of the reported international range for non-persistence and overall discontinuation, which may in part reflect the use of a prevalent rather than an incident cohort that overrepresents patients with longer treatment duration, as well as the benefits of universal healthcare coverage in reducing access-related barriers [15, 24–29]. However, the dramatic institutional variability we observed suggests that healthcare system structure alone is insufficient to ensure optimal treatment continuity.

Recent comprehensive analyses of barriers to nAMD care identify seven major categories: travel burden, psychological barriers, financial burden, treatment regimen challenges, comorbidities, provider-level factors, and system-level barriers. Switzerland’s universal healthcare system theoretically mitigates financial barriers, yet our institutional variability findings suggest that other barrier categories, particularly provider-level and system-level factors, remain significant determinants of treatment outcomes [20, 26, 29, 30].

Contemporary chronic care approaches in other medical specialties emphasise systematic risk stratification and personalised intervention matching [31]. However, ophthalmology has been slow to adopt such integrated chronic care models despite mounting evidence that retinal diseases require long-term management strategies similar to other chronic conditions [32]. Recent initiatives provide structured frameworks for identifying and addressing specific adherence barriers through systematic patient risk profiling [33, 34]. This approach recognises that non-persistence stems from diverse, patient-specific barriers including anxiety and depression, caregiver burden, cognitive challenges, travel difficulties, and access limitations, each requiring tailored interventions.

Our finding that patients utilising digital hyperacuity home monitoring showed an association with reduced treatment discontinuation highlights digital home monitoring as a promising strategy to address non-persistence. This aligns with growing evidence that digital health interventions can improve chronic disease management through enhanced patient engagement and real-time monitoring capabilities [34]. The mechanisms underlying improved persistence likely include increased patient empowerment, early detection of visual changes, and sustained engagement between clinic visits. However, home monitoring users differed from non-users at baseline, being younger and having better initial visual acuity; although we adjusted for key baseline covariates, residual confounding due to unmeasured factors cannot be excluded.

### Strengths and limitations

Several key features distinguish this investigation. The large, nationwide sample and integration of granular clinical data with population-based insurance claims allowed for a nuanced evaluation of variability in treatment discontinuation at both institutional and national levels. Linking these datasets enabled precise validation of discontinuation events, ensuring that cases identified in the clinical cohort truly reflected treatment cessation rather than transfer of care, which was a scenario found to be rare. The ability to differentiate supervised from unsupervised discontinuation further provided clinical context to patient trajectories. Importantly, the dual-dataset approach facilitated robust assessment of population-level discontinuation trends and combined with analysis of home monitoring engagement, offered novel insights into the possible value of digital health tools for enhancing persistence.

This study is not without limitations. The retrospective observational design precludes causal inference and, in the claims dataset, visual outcome information is lacking. We were unable to ascertain specific reasons for treatment discontinuation, and the Helsana data could not differentiate nAMD from other chorioretinal disorders except for DMO. The definition applied for DMO prioritised sensitivity, which may have resulted in misclassification of other chorioretinal conditions as DMO. Claims data analyses may have overestimated treatment duration due to the inability to distinguish injections by laterality.

The substantial inter-centre heterogeneity in discontinuation proportions likely reflects differences in physician practice patterns, patient demographics, access barriers, and disease characteristics across centres. However, our claims-based dataset does not capture visual acuity or treatment regimen protocols, limiting our ability to definitely identify the drivers of this variation. Future prospective studies with detailed centre-level documentation would be valuable for understanding which system-level factors most influence treatment discontinuation patterns.

The voluntary uptake of home monitoring may have introduced selection bias and therefore influenced the observed association between home monitoring and non-persistence. Although we adjusted for key baseline covariates, we cannot rule out bias from unmeasured differences between users and non-users. Future studies in larger cohorts, potentially using target trial emulation approaches, are needed to further investigate the association of home monitoring on persistence.

### Implications for research

Future investigations should prioritise development and validation of systematic risk stratification tools that can identify patients at high risk for non-persistence early in their treatment journey. Such tools should integrate multiple risk domains including psychosocial factors (anxiety, depression), practical barriers (transportation, caregiver availability), disease understanding, and treatment burden perceptions. Standardised patient-reported outcome measures (PROMs) specifically designed for adherence barrier assessment require broader validation and implementation [35].

Prospective research examining institutional-level interventions to reduce discontinuation variability represents a critical priority. Such studies should evaluate systematic quality improvement initiatives including standardised patient education protocols, care coordinator programmes, and integrated chronic care models adapted from successful approaches in diabetes and oncology care.

### Implications for practice

Clinicians must recognise that unsupervised discontinuation represents a substantial and often undetected threat to patient outcomes. Our findings suggest that systematic screening for adherence barriers should become standard practice, utilising structured assessment tools to identify patients at risk for non-persistence. The dramatic institutional variability we observed indicates that treatment persistence is highly modifiable through systematic quality improvement initiatives. We advocate for implementation of integrated chronic care approaches adapted from successful models in other specialties, incorporating patient journey coordinators, systematic risk profiling, and matched interventions for identified barriers. Digital home monitoring may be considered to increase patient participation. Enhanced patient education emphasising the chronic nature of nAMD and realistic treatment expectations represent a fundamental requirement, as patient understanding of disease chronicity remains a critical determinant of long-term adherence.

Healthcare systems should consider pilot programmes to evaluate real-world effectiveness and develop optimal integration protocols. Current initiatives such as the AMD Barometer programme provide valuable frameworks for systematic barrier assessment and should be considered for broader adoption.

Critically, ophthalmology must embrace chronic care management principles that have proven successful in other specialties, moving beyond the traditional acute-care model to comprehensive, longitudinal patient engagement strategies that address the multifaceted nature of treatment persistence challenges.

## Conclusions

Anti-angiogenic therapy for nAMD should generally be continued lifelong, as discontinuation carries substantial risk of disease reactivation and vision loss. However, our real-world data reveal that treatment discontinuation is common and highly variable across institutions, indicating significant opportunities for systematic quality improvement. The institutional heterogeneity points to substantial variation in both guideline adoption and care delivery models across Swiss AMD providers. This suggests that optimising adherence to evidence-based protocols, alongside interventions tailored to address patient-specific barriers, holds potential for improving treatment persistence and outcomes.

The path forward requires ophthalmology to adopt proven chronic care management strategies, systematic risk stratification approaches, and integrated digital health solutions. By implementing comprehensive patient engagement programmes that address the diverse barriers to treatment persistence, we can significantly improve real-world outcomes and reduce the substantial burden of preventable vision loss in nAMD patients.

## Summary

### What was known before

- Although anti-angiogenic therapies have demonstrated robust efficacy in clinical trials, real-world outcomes remain consistently inferior.
- Treatment discontinuation represents a key barrier to sustained visual benefit and encompasses two distinct phenomena: non-persistence and planned discontinuation.
- Switzerland’s universal healthcare system offers a unique setting to investigate these patterns, free from the access barriers typically associated with insurance coverage in other contexts.

### What this study adds

- Our findings show that unsupervised discontinuation and complete disengagement from ophthalmologic care in nAMD is substantial and grows over time.
- Discontinuation rates varied nearly 15-fold across the 20 largest Swiss treatment centres.
- The findings underscore substantial institutional heterogeneity and the potential for targeted, systematic, patient-centred interventions.

## Supplementary information

Supplemental Material

Eye reporting checklist

## Data Availability

The datasets generated during and/or analysed during the current study are available from the corresponding author on reasonable request.
