# Supplementary material for: Prevalence and patterns of anti-angiogenic treatment discontinuation in neovascular age-related macular degeneration—insights from health claims data
Source: Eye (Lond). 2026 Feb 24;40(7):972–8. doi: 10.1038/s41433-026-04333-3 (PMC13161405; doi:10.1038/s41433-026-04333-3)
Supplement: Supplementary file 1 [file 41433_2026_4333_MOESM2_ESM.pdf]

Corresponding Author Name:_Bachmann
Manuscript Number: EYE-25-2739RR

Reporting Checklist

This checklist is used to ensure good reporting standards and to improve the reproducibility of published results. **Please respond completely to all questions relevant to your manuscript, indicating "N/A" if the question does not apply.** For more information, please read the journal's Guide to Authors.

☐ Check here to confirm that the following information is available in the Material & Methods section:

x the **exact sample size (*n*)** for each experimental group/condition, given as a number, not a range;

x a **description of the sample collection** allowing the reader to understand whether the samples represent **technical or biological replicates** (including how many animals, litters, culture, etc.);

x a **statement of how many times the experiment shown was replicated in the laboratory**;

x **definitions of statistical methods and measures**: (For small sample sizes (n<5) descriptive statistics are not appropriate, instead plot individual data points)

x
- very common tests, such as *t*-test, simple χ^2 tests, Wilcoxon and Mann-Whitney tests, can be unambiguously identified by name only, but more complex techniques should be described in the methods section;
- are tests one-sided or two-sided?
- are there adjustments for multiple comparisons?

x
- **statistical test results**, e.g., ***P* values**;
- definition of **'center values'** as **median or mean**;
- definition of **error bars as s.d. or s.e.m. or c.i.**

Please ensure that the answers to the following questions are reported **in the manuscript itself.** We encourage you to include a specific subsection in the methods section for statistics, reagents and animal models. Below, provide the page number or section and paragraph number.

**Statistics and general methods**	**Reported in section/paragraph or page #**
1. How was the sample size chosen to ensure adequate power to detect a pre-specified effect size? (Give section/paragraph or page #)	we took all available patients. No formal sample size calculation was therefore performed
For animal studies, include a statement about sample size estimate even if no statistical methods were used.	not appicable
2. Describe inclusion/exclusion criteria if samples or animals were excluded from the analysis. Were the criteria pre-established? (Give section/paragraph or page #)	lines 115-167
3. If a method of randomization was used to determine how samples/animals were allocated to experimental groups and processed, describe it. (Give section/paragraph or page #)	not applicable
For animal studies, include a statement about randomization even if no randomization was used.	not applicable

4. If the investigator was blinded to the group allocation during the experiment and/or when assessing the outcome, state the extent of blinding. (Give section/paragraph or page #)	lines 168-177
For animal studies, include a statement about blinding even if no blinding was done.	not applicable
5. For every figure, are statistical tests justified as appropriate?	Figures: just descriptive
Do the data meet the assumptions of the tests (e.g., normal distribution)?	lines 208-219
Is there an estimate of variation within each group of data?	yes
Is the variance similar between the groups that are being statistically compared? (Give section/paragraph or page #)	Results section, first paragraph

**Reagents**	**Reported in section/paragraph or page #**
6. Report the source of antibodies (vendor 7. and catalog number)	not applicable
8. Identify the source of cell lines and report if they were recently authenticated (e.g., by STR profiling) and tested for mycoplasma contamination	not applicable

**Animal Models**	**Reported in section/paragraph or page #**
8. Report species, strain, sex and age of animals	
9. For experiments involving live vertebrates, include a statement of compliance with ethical regulations and identify the committee(s) approving the experiments.	

10. We recommend consulting the ARRIVE guidelines (*PLoS Biol.* **8**(6), e1000412,2010) to ensure that other relevant aspects of animal studies are adequately reported.

Human subjects

	Reported in section/paragraph or page #
11. Identify the committee(s) approving the<br>12. study protocol.	221-228
13. Include a statement confirming that informed consent was obtained from all subjects.	226
14. For publication of identifiable patient photos, include a statement confirming that consent to publish was obtained. This is not required for photos just of the eye which do not identify the patient.	N/A
15. Report the clinical trial registration number (at ClinicalTrials.gov or equivalent).	N/A

16. For phase II and III randomized controlled trials, please refer to the CONSORT statement and submit the CONSORT checklist with your submission.

17. For tumor marker prognostic studies, we recommend that you follow the REMARK reporting guidelines.
