# Supplementary material for: Prevalence and patterns of anti-angiogenic treatment discontinuation in neovascular age-related macular degeneration—insights from health claims data
Source: Eye (Lond). 2026 Feb 24;40(7):972–8. doi: 10.1038/s41433-026-04333-3 (PMC13161405; doi:10.1038/s41433-026-04333-3)
Supplement: Supplementary file 2 [file 41433_2026_4333_MOESM1_ESM.docx]

**Supplementary Material**

**Supplementary Material 1 – Clinical Dataset (LUKS) Details**

Patient-level information

- Age at baseline
- Gender (male/female)
- Diagnosis (e.g., AMD, DMO, CRVO, other)
- Relevant ocular and systemic history (e.g., prior diagnoses, comorbidities)

Eye-level information

- Affected eye (right/left, or bilateral involvement)
- History of diagnosis per eye
- Treatment history per eye (intravitreal injections, prior ocular procedures)

Visit-level information (recorded per visit and per eye)

- Date of visit
- Best-corrected visual acuity (ETDRS letters)
- OCT: Central retinal thickness (in mm)
- Intravitreal injection(s): date, drug name, dose administered

Home-monitoring subset (where available)

- Date and time of home-monitoring sessions
- Duration of testing (overall and per subtask)
- Test performance details (overall completion, subtasks)

**Supplementary Material 2 – Insurance Claim Dataset (Helsana AG) Details**

Cohort inclusion criteria

- Intravitreal injection (IVI): evidence of treatment with ≥1 of the following preparations:
  - Avastin (Swiss Pharmacode: 2918877, 2918914; additional criterion required due to off-label use: ≥1 same-day billing entry with Tarmed tariff code 08.XXXX)
  - Lucentis (Swiss Pharmacode: 5907288, 6504552, 6063496)
  - Eylea (Swiss Pharmacode: 7755254, 5412919)
  - Eylea High-Dose (Swiss Pharmacode: 1100478)
  - Vabysmo (Swiss Pharmacode: 7838791)
- At least 1 IVI treatment day in 2018
- At least 4 IVI treatment days documented
- At least 3 treatment days within <365 days

Patient-level variables

- Demographics: age, gender
- Insurance information:
  - Deductible (annual, in Swiss Francs)
  - Insurance model (Standard model; alternative insurance model, including General practitioner gatekeeping, Health Maintenance Organization (HMO) and Telemedicine model)
  - Insurance coverage changes during follow-up
- Vital status: date of death (if applicable)

Treatment-related variables

- IVI information: date of injection, preparation used, dose
- Number and timing of IVI treatment days

**Supplementary Material 3 – Aggregation of Clinics/Practices in Regions**

Regions according to the Swiss Federal Statistical Office^1^

-	LEM – Région lémanique (Lake Geneva region)

-	MIT – Espace Mittelland (Central Mittelland)

-	NW – Nordwestschweiz (Northwestern Switzerland)

-	OST – Ostschweiz (Eastern Switzerland)

-	TI – Ticino (Ticino)

-	ZH – Zürich (Zurich)

-	ZS – Zentralschweiz (Central Switzerland)

^1^Federal Statistical Office (FSO). Analyseregionen. Neuchâtel: Federal Statistical Office; accessed September 2025.

Available from:

https://www.bfs.admin.ch/bfs/de/home/statistiken/querschnittsthemen/raeumliche-analysen/raeumliche-gliederungen/analyseregionen.html

**Supplementary Table 1** – **Clinical Characteristics of Patients Treated at the Cantonal Hospital Lucerne: Overall and Stratified by Home-Monitoring Use: AMD Subset**

| **Characteristic** | **Overall**  n = 1,387*^1^* | **Home-Monitoring** | | **p-value** |
| --- | --- | --- | --- | --- |
|  |  | No  n = 1,287*^1^* | Yes  n = 100*^1^* |  |
| **Age** | 77 (9) | 77 (9) | 73 (8) | <0.001 |
| **Sex** |  |  |  | 0.08 |
| F | 877 (63%) | 822 (64%) | 55 (55%) |  |
| M | 510 (37%) | 465 (36%) | 45 (45%) |  |
| **Initial visual acuity [letters]** ^2^ | 64 (17) | 63 (17) | 71 (13) | <0.001 |
| **Initial OCT (central retinal thickness [mm])** ^3^ | 379 (122) | 381 (124) | 361 (101) | 0.3 |
| **Bilateral injections** |  |  |  | >0.9 |
| No | 701 (51%) | 651 (51%) | 50 (50%) |  |
| Yes | 686 (49%) | 636 (49%) | 50 (50%) |  |
| **Injections count** | 39 (27) | 38 (22) | 52 (32) | <0.001 |

^1^Mean (standard deviation [SD]); ^2^ initial visual acuity missing for 67 patients; ^3^ initial OCT value missing for 52 patients; n (%). AMD: age-related macular degeneration. DMO: diabetic macular oedema.

**Supplementary Table 2** – Overview of Patient Characteristics Across Centres/Regions

| **Characteristic** | **Centre 1** | **Centre 2** | **Centre 3** | **Centre 4** | **Centre 5** | **Centre 6** | **Centre 7** | **Centre 8** | **Centre 9** | **Centre 10** | **Centre 11** | **Centre 12** | **Centre 13** | **Centre 14** | **Centre 15** | **Centre 16** | **Centre 17** | **Centre 18** | **Centre 19** | **Centre 20** | **ZH** | **MIT** | **OST** | **LEM** | **NW** | **TI** | **ZS** |
| --- | --- | --- | --- | --- | --- | --- | --- | --- | --- | --- | --- | --- | --- | --- | --- | --- | --- | --- | --- | --- | --- | --- | --- | --- | --- | --- | --- |
| **Patients, n** | 575 | 304 | 269 | 274 | 235 | 175 | 171 | 160 | 150 | 153 | 164 | 119 | 99 | 70 | 67 | 64 | 65 | 60 | 53 | 54 | 360 | 338 | 303 | 249 | 135 | 100 | 74 |
| **Age, mean (SD)** | 79 (10) | 77  (9) | 75 (11) | 76 (12) | 76 (11) | 78 (10) | 74 (11) | 76 (10) | 75 (13) | 75 (10) | 75 (12) | 78  (9) | 79  (9) | 79 (10) | 78 (11) | 78  (8) | 77 (10) | 77  (9) | 78 (13) | 72 (14) | 79 (10) | 79  (9) | 77  (9) | 77 (11) | 79  (8) | 79  (7) | 79  (9) |
| **Gender** |  |  |  |  |  |  |  |  |  |  |  |  |  |  |  |  |  |  |  |  |  |  |  |  |  |  |  |
| Female, n (%) | 357 (62%) | 180 (59%) | 164 (61%) | 146 (53%) | 131 (56%) | 107 (61%) | 99 (58%) | 101 (63%) | 96 (64%) | 85 (56%) | 95 (58%) | 84 (71%) | 67 (68%) | 50 (71%) | 40 (60%) | 46 (72%) | 43 (66%) | 34 (57%) | 28 (53%) | 26 (48%) | 225 (63%) | 213 (63%) | 188 (62%) | 162 (65%) | 84 (62%) | 61 (61%) | 39 (53%) |
| Male, n (%) | 218 (38%) | 124 (41%) | 105 (39%) | 128 (47%) | 104 (44%) | 68 (39%) | 72 (42%) | 59 (37%) | 54 (36%) | 68 (44%) | 69 (42%) | 35 (29%) | 32 (32%) | 20 (29%) | 27 (40%) | 18 (28%) | 22 (34%) | 26 (43%) | 25 (47%) | 28 (52%) | 135 (38%) | 125 (37%) | 115 (38%) | 87 (35%) | 51 (38%) | 39 (39%) | 35 (47%) |
| **Deductible** |  |  |  |  |  |  |  |  |  |  |  |  |  |  |  |  |  |  |  |  |  |  |  |  |  |  |  |
| Low (300-500 Swiss Francs) | 528 (92%) | 285 (94%) | 241 (90%) | 254 (93%) | 217 (92%) | 159 (91%) | 157 (92%) | 151 (94%) | 130 (87%) | 141 (92%) | 156 (95%) | 107 (90%) | 90 (91%) | 66 (94%) | 62 (93%) | 59 (92%) | 60 (92%) | 51 (85%) | 51 (96%) | 48 (89%) | 320 (89%) | 315 (93%) | 273 (90%) | 238 (96%) | 129 (96%) | 92 (92%) | 68 (92%) |
| High (1000-2500 Swiss Francs) | 47 (8%) | 19 (6%) | 28 (10%) | 20 (7%) | 18 (8%) | 16 (9%) | 14 (8%) | 9  (6%) | 20 (13%) | 12 (8%) | 8  (5%) | 12 (10%) | 9  (9%) | 4  (6%) | 5  (8%) | 5  (8%) | 5  (8%) | 9 (15%) | 2  (4%) | 6 (11%) | 40 (11%) | 23 (7%) | 30 (10%) | 11 (4%) | 6  (4%) | 8  (9%) | 6  (8%) |
| **Insurance model** |  |  |  |  |  |  |  |  |  |  |  |  |  |  |  |  |  |  |  |  |  |  |  |  |  |  |  |
| Standard model, n (%) | 345 (60%) | 153 (50%) | 120 (45%) | 149 (54%) | 102 (43%) | 69 (39%) | 100 (58%) | 92 (58%) | 103 (69%) | 69 (45%) | 73 (45%) | 67 (56%) | 59 (60%) | 32 (46%) | 23 (34%) | 25 (39%) | 29 (45%) | 36 (60%) | 37 (70%) | 34 (63%) | 197 (55%) | 177 (52%) | 135 (45%) | 161 (65%) | 70 (52%) | 49 (49%) | 46 (62%) |
| Alternative insurance models (including GP gatekeeping, HMO, and telemedicine models) | 230 (40%) | 151 (50%) | 149 (55%) | 125 (46%) | 133 (57%) | 106 (61%) | 71 (42%) | 68 (43%) | 47 (31%) | 84 (55%) | 91 (55%) | 52 (44%) | 40 (40%) | 38 (54%) | 44 (66%) | 39 (61%) | 36 (55%) | 24 (40%) | 16 (30%) | 20 (37%) | 163 (45%) | 161 (48%) | 168 (55%) | 88 (35%) | 65 (48%) | 51 (51%) | 28 (38%) |
| **Diagnosis** |  |  |  |  |  |  |  |  |  |  |  |  |  |  |  |  |  |  |  |  |  |  |  |  |  |  |  |
| AMD and other retinal diseases, n (%) | 455 (79%) | 233 (77%) | 213 (79%) | 197 (72%) | 184 (78%) | 138 (79%) | 132 (77%) | 124 (78%) | 115 (77%) | 112 (73%) | 109 (66%) | 102 (86%) | 73 (74%) | 57 (81%) | 56 (84%) | 55 (86%) | 43 (66%) | 42 (70%) | 35 (66%) | 21 (39%) | 293 (81%) | 268 (79%) | 242 (80%) | 183 (74%) | 102 (76%) | 72  (75%) | 58 (78%) |
| DMO, n (%) | 120 (21%) | 71 (23%) | 56 (21%) | 77 (28%) | 51 (22%) | 37 (21%) | 39 (23%) | 36 (23%) | 35 (23%) | 41 (27%) | 55 (34%) | 17 (14%) | 26 (26%) | 13 (19%) | 11 (16%) | 9 (14%) | 22 (34%) | 18 (30%) | 18 (34%) | 33 (61%) | 67 (19%) | 70 (21%) | 61 (20%) | 66 (27%) | 33 (24%) | 25 (25%) | 16 (22%) |
| **Treatment duration in days, mean (SD)** | 1960 (902) | 1866 (903) | 1818 (968) | 1779 (894) | 1822 (907) | 1525 (700) | 2276 (822) | 1937 (939) | 2040 (949) | 1964 (885) | 1790 (953) | 1974 (901) | 1804 (885) | 1536 (704) | 1565 (773) | 2000 (937) | 1475 (850) | 2148 (792) | 1256 (750) | 1942 (954) | 1586 (866) | 1793 (852) | 1804 (877) | 1628 (825) | 1759 (792) | 1545 (926) | 1725 (904) |

AMD: age-related macular degeneration. DMO: diabetic macular oedema. GP = General Practitioner. HMO = Health Maintenance Organization. SD: standard deviation. Regions: LEM = Région lémanique (Lake Geneva region); MIT = Espace Mittelland (Central Mittelland); NW = Nordwestschweiz (Northwestern Switzerland); OST = Ostschweiz (Eastern Switzerland); TI = Ticino (Ticino); ZH = Zürich (Zurich); ZS = Zentralschweiz (Central Switzerland)
